# Renal medullary carcinoma masquerading as renal infection: a case report

**DOI:** 10.1186/s12882-020-01730-y

**Published:** 2020-03-05

**Authors:** Zhong-Ming Huang, Hai Wang, Zhi-Gang Ji

**Affiliations:** Department of Urology, Peking Union Medical College Hospital, Chinese Academy of Medical Science and Peking Union Medical College, No.1 Shuaifuyuan Wangfujing, Dongcheng, Beijing, 100730 China

**Keywords:** Renal medullary carcinoma, Fever, Hematuria, Differential diagnosis

## Abstract

**Background:**

Renal medullary carcinoma is a rare and aggressive tumor and often seen in young adults with sickle cell hemoglobinopathies.

**Case presentation:**

We report a case of renal medullary carcinoma in a 29-year old male patient with an occupying renal lesion who presented with fever, flank pain and hematuria. The patient received intensive antibiotics treatment, but no improvement was seen. The symptoms disappeared after laparoscopic radical left nephrectomy. Postoperative pathological study showed that the mass was renal medullary carcinoma.

**Conclusions:**

Our case suggests that renal medullary carcinoma should be considered in differential diagnoses of patients with occupying renal lesions who have fever of unknown origin.

## Background

Renal medullary carcinoma is a rare and aggressive tumor and nearly uniformly lethal [1–3]. The disease occurs almost exclusively in adolescents and young adults with sickle cell hemoglobinopathies [4]. Renal medullary carcinoma is characterized by gross hematuria, abdominal or flank pain, and loss of weight, which may lead erroneously to a diagnosis of renal infections or abscess. Therefore, early diagnosis of renal medullary carcinoma requires a high level of suspicion [3, 5]. It has been recommended that renal medullary carcinoma should be considered in patients less than 50 years of age with poorly differentiated carcinoma that arises from the renal medulla [3] and renal medullary carcinoma patients should be tested for sickle cell hemoglobinopathies. Rarely, renal medullary carcinoma may be concomitant with renal echinococcosis. Furthermore, because the disease exhibits aggressive growth and is typically at an advanced stage upon presentation, patients with renal medullary carcinoma have a poor survival [6]. We report a case of renal medullary carcinoma in a 29-year old male patient who presented to our hospital with fever, flank pain and hematuria refractory to antibiotics therapy.

## Case report

A 29-year old man presented on January 7, 2019 with dry cough and fever with shivering accompanied by left flank pain for over 40 days and a left kidney occupying lesion for 10 days. The patient developed dry cough without apparent trigger and fever (temperature 37.7 °C) in the afternoon, with shivering accompanied by left flank pain. On November 30, 2018, blood chemistries at a local hospital showed increased erythrocyte sedimentation rate (ESR) and C-reactive protein (CRP) and urinalysis revealed hematuria and proteinuria and he received intravenous metronidazole and penicillin for 3 days without apparent improvement. On December 5, 2018, he received intravenous levofloxacin at a provincial hospital for 2 days; left flank pain was worsened with fever (38.7 °C) with shivering during the treatment. Abdominal MRI scan revealed left kidney enlargement with a hypointense shadow with peripheral enhancement. His symptoms did not abate despite intensive antibiotics with piperacillin plus tazobactam, etimicin, linezolid, voriconazole, biapenem, and sulperazone. No symptomatic improvement occurred. The patient had lost about 5 kg in body weight since his illness.

The patient had a history of acute lymphoblastic leukemia and received allogeneic stem cell transplantation in 2007. He did not experience an acute kidney injury episode, a renal infarct or any renal disease in the past.

**Fig. 1:**
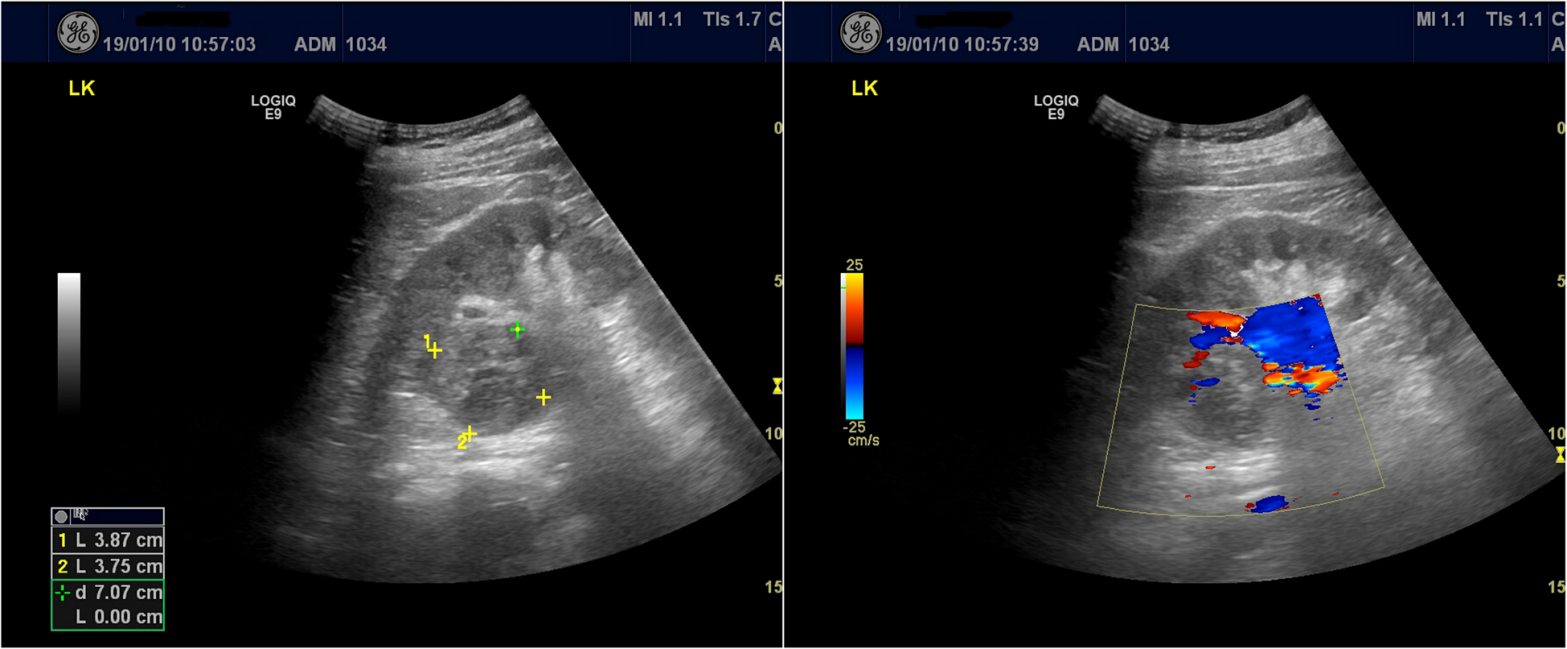
Doppler flow imaging ultrasonography (CDFI) in a 29-year old man shows a regular hypoechoic mass, 3.8×3.7 cm in size, with distinct borders in the upper pole of the left kidney and dot or strip-like flow signal

Admission examination revealed a temperature of 37.7 °C and a pulse of 107/min. No other remarkable physical findings were seen. His blood chemistries showed white blood cell (WBC) counts at 12.3 × 10^9^/L with 63.7% neutrophils and hsCRP 125.20 mg/L, ferritin 1421 ng/mL and ESR 104 mm/h. Serum creatinine was 97 μmol/L and blood urea nitrogen (BUN) was 9.04 mmol/L. Routine urinary tests on six occasions were unremarkable and showed no signs of infection. Doppler flow imaging ultrasonography (CDFI) showed a regular hypoechoic mass with distinct borders in the upper pole of the left kidney, 3.8 × 3.7 cm in size, and dot or strip-like flow signal (Fig. 1). Biopsy pathology on January 11, 2019 showed scant left kidney necrosis. A tentative diagnosis of fever of unknown origin and probable left kidney infection was made.

**Fig. 2:**
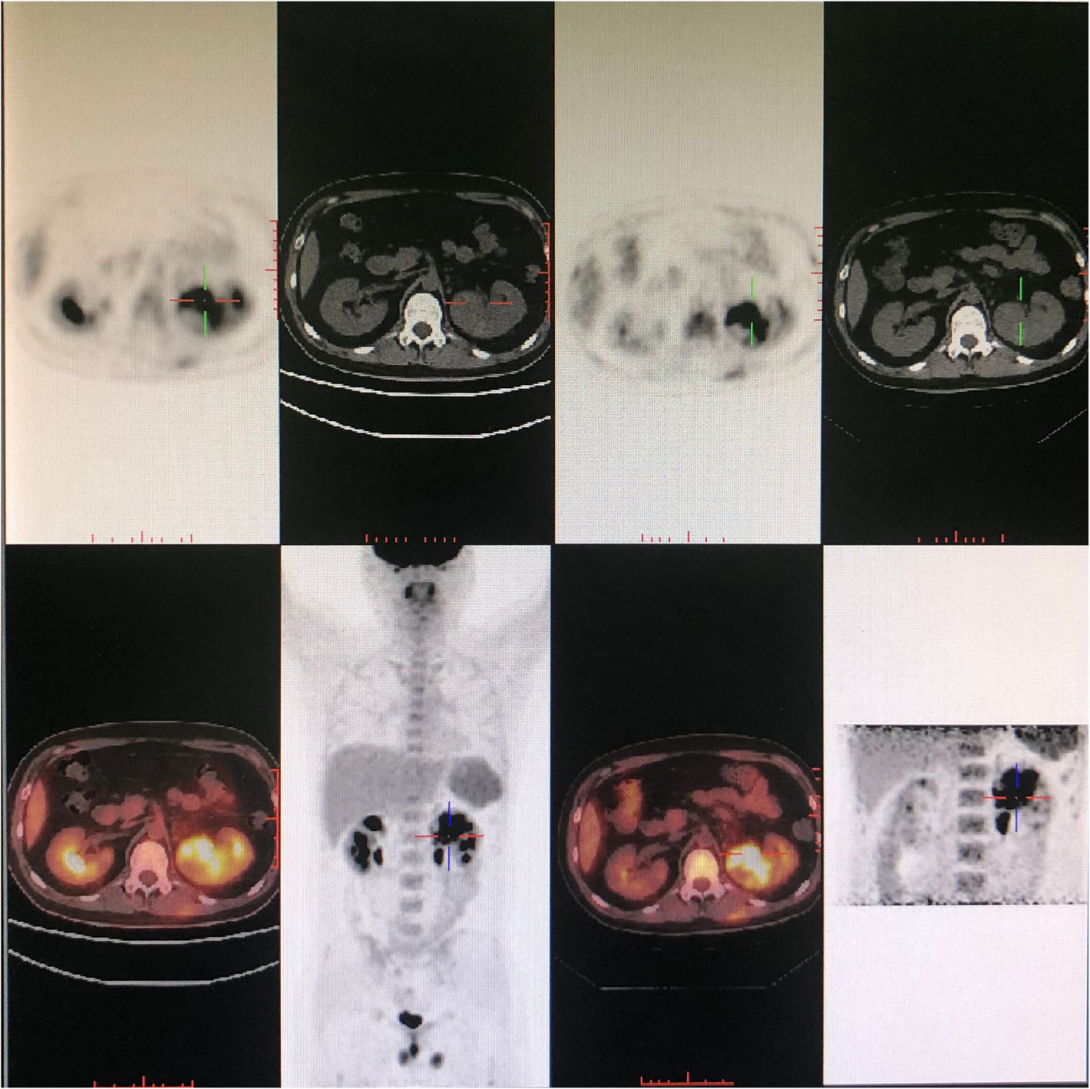
PET/CT scan on reveals an occupying lesion, approximately 4.7 × 5.7 × 5.1 cm in size, in the upper pole of the left kidney. The early SUVmax is 14.5 and the delayed SUVmax is 21.7. The upper segment of the left ureter is thickened and occluded

The patient received intravenous imipenem (1 g every 8 h) and indomethacin. Imipenem was replaced with intravenous meropenem (1 g every 8 h) due to gastrointestinal side effect. Levofloxacin (0.5 g once daily) was added on January 18, 2019. However, fever persisted. PET/CT scan on January 20, 2019 revealed an occupying lesion, approximately 4.7 × 5.7 × 5.1 cm in size, in the upper pole of the left kidney (Fig. 2). The early SUVmax was 14.5 and delayed SUVmax was 21.7. The upper segment of the left ureter was thickened and occluded. Laboratory study on January 26, 2019 showed WBC counts at 14.22 × 10^9^/L, neutrophils 8.45 × 10^9^/L, alanine transferase (ALT) 152 U/L, hsCRP 146.0 mg/L and ESR 121 mm/h. Serum creatinine was 61 μmol/L and BUN was 5.78 mmol/L. Considering inefficacy of antibiotics therapy and elevations in ALT, antibiotics were discontinued. Renal malignancy was considered.

**Fig. 3:**
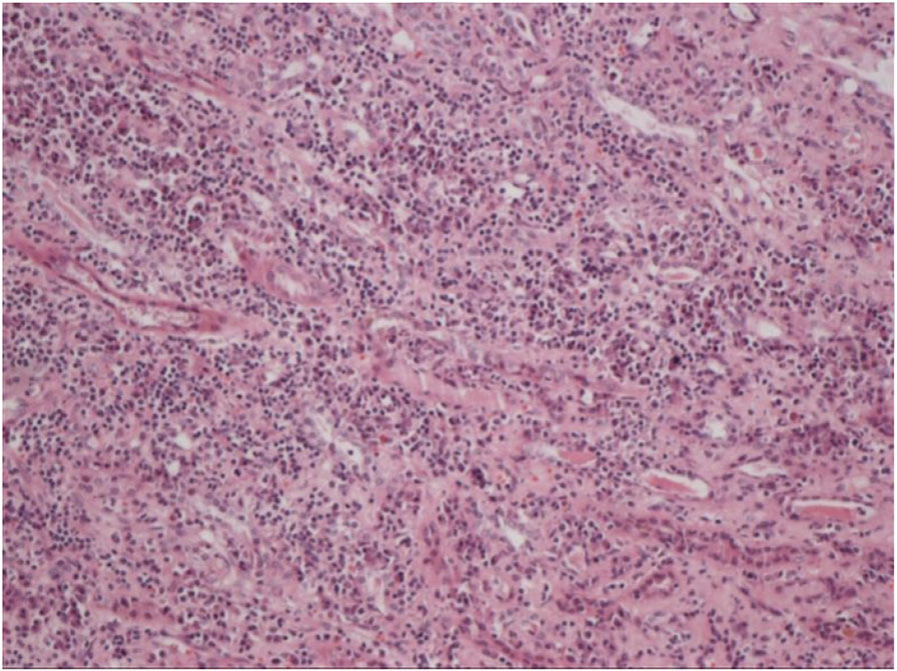
Immunohistochemistry of the resected left suprarenal mass shows that the tumor is positive for vimentin and partially positive for CK19, PAX-8, and P63 and weakly positive for CAM5.2 and sparsely positive for S-100. The Ki-67 index is 50%

The patient underwent after laparoscopic radical left nephrectomy under general anesthesia on February 2, 2019. Intraoperatively, a solid mass, 5 cm ╳ 4.5 cm ╳ 2.5 cm in size, was observed in the upper pole of the left kidney. The cut surface appeared beige brown and the tumor margin was 0.5 cm from the renal pelvis. Grossly, the tumor mass invaded the renal capsule. No obviously enlarged lymph nodes were palpitated. The body temperature became normal (36.3) on the first postoperative day and postoperative ALT was 63 U/L. Postoperative pathology suggested renal medullary carcinoma (Fig. 3) and immunohistochemistry revealed CK19 (partially+), PAX-8 (partially+), S-100 (scattered +), HMB45 (−), vimentin (+), CK7(−), AE1/AE3(+), CEA(−), CD10 (weakly +), Ki67 (index 50%), OCT3/4 (−), CAM5.2 (weakly +), and p63 (partially+) (Fig. 3). His serum creatinine was 90 μmol/L and BUN was 6.69 mmol/L on February 9, 2019. The patient was discharged from the hospital on February 11, 2019. The patient was followed up every 3 months and no tumor recurrence or metastasis occurred at the final follow up at 9 months.

## Discussion and conclusions

Renal medullary carcinoma is an uncommon and aggressive disease. The tumor causes nonspecific manifestations in the patient including flank pain, hematuria and loss of body weight [3, 7]. These nonspecific symptoms were also present in our patient. Often, in the absence of a high index of suspicion, lack of specific manifestations leads to erroneous diagnoses such as collecting duct carcinoma and non-neoplastic diseases. One prominent symptom of our current case was persistent fever despite intensive antibiotics therapy. Though renal occupying lesion was detected by abdominal ultrasound imaging and subsequently by PET/CT scan, because of the non-specific manifestations including fever, flank pain and hematuria, infection was considered to be the most likely source of the patient’s symptoms, leading to delays diagnosis and treatment. Leukocytosis and increased inflammatory markers such as CRP and ESR also led the physicians to consider infection as the underlying cause of the patient’s manifestations.

Renal medullary carcinoma is more commonly found in young adults with sickle cell hemoglobinopathies [1]. Hemoglobin analysis showed no sickle cell hemoglobinopathies in our case. This is consistent with a report on 6 Chinese patients with renal medullary carcinoma; only one patient was found to have sickle cell hemoglobinopathy [8]. Sickle cell hemoglobinopathies are uncommon in Han Chinese and Chinese renal medullary carcinoma patients tend to be older than patients of African descent. In the series by Shi et al. [8], the age of the 6 Chinese patients ranged from 22 to 72 years (median 56.5 years).

Though imaging studies including ultrasonography and PET/CT scan were performed in our patient, the findings were suggestive of the presence of renal tumors, but they were inconclusive and cannot distinguish between renal infection and renal medullary carcinoma and other renal neoplastic diseases. The persistence of the patient’s symptoms despite intensive antibiotics therapy led the physicians to explore alternative diagnosis and the decision to proceed with laparoscopic radical left nephrectomy. Currently, the level of evidence for renal medullary carcinoma is low and is based mostly on anecdotal case reports and case series. Given the rarity of the condition, no clinical trial data is available. The renal medullary carcinoma Working Group recommends upfront radical nephrectomy in renal medullary carcinoma patients with good performance status and low metastatic burden [3]. Fever and flank pain in our patient disappeared after left radical nephrectomy and there was no tumor recurrence or metastasis at the final follow up visit, suggesting effectiveness of radical nephrectomy for renal medullary carcinoma. Our patient did not receive chemotherapy or angiogenesis inhibitors. Shah et al. [4]*.* studied 52 renal medullary carcinoma patients collected over a 15-year period from multiple institutions and showed that patients undergoing nephrectomy had a longer survival than those receiving systemic therapy alone, was associated with longer survival (16.4 vs. 7.0 months) and 28 patients receiving vascular endothelial growth factor (EGFR) inhibitors had no objective response.

Renal medullary carcinoma is a rare disease with non-specific clinical manifestations. Few cases have been documented in patients of Han Chinese descent. When an occupying lesion is detected, without pathological evidence, physicians tend to consider more common renal tumors in their diagnosis. In our case, renal medullary carcinoma was masquerading as renal infection with prominent fever and flank pain, leading to erroneous diagnosis and delayed treatment. Our case suggests that renal medullary carcinoma should be considered in differential diagnoses of patients with occupying renal lesions who have fever of unknown origin.

## Data Availability

The datasets generated and analyzed during the current study are available from the corresponding author on reasonable request.

## Abbreviations

ALT: Alanine transferase
CDFI: Color Doppler flow image.
CRP: C-reactive protein.
EGFR: Endothelial growth factor receptor.
ESR: Erythrocyte sedimentation rate.
WBC: White blood cell.
